# Human Prostate Epithelial Cells Activate the AIM2 Inflammasome upon Cellular Senescence: Role of POP3 Protein in Aging-Related Prostatic Inflammation

**DOI:** 10.3390/life11040366

**Published:** 2021-04-20

**Authors:** Ravichandran Panchanathan, Vaikundamoorthy Ramalingam, Hongzhu Liu, Divaker Choubey

**Affiliations:** 1 Department of Environmental Health, University of Cincinnati, Cincinnati, OH 45221, USA; pancharn@ucmail.uc.edu (R.P.); ramalingam@iict.res.in (V.R.); liuhongzhu1@hotmail.com (H.L.); 2 Centre for Natural Products and Traditional Knowledge, CSIR—Indian Institute of Chemical Technology, Hyderabad 500 007, India

**Keywords:** prostate, senescence, inflammation, AIM2 inflammasome, POP3

## Abstract

Increased levels of type I (T1) interferon (IFN)-inducible POP3 protein in myeloid cells inhibit activation of the AIM2 inflammasome and production of IL-1β and IL-18 proinflammatory cytokines. The *AIM2* mRNA levels were significantly higher in benign prostate hyperplasia (BPH) than the normal prostate. Further, human normal prostate epithelial cells (PrECs), upon becoming senescent, activated an inflammasome. Because in aging related BPH senescent PrECs accumulate, we investigated the role of POP3 and AIM2 proteins in pre-senescent and senescent PrECs. Here we report that the basal levels of the POP3 mRNA and protein were lower in senescent (*versus* young or old) PrECs that exhibited activation of the T1 IFN response. Further, treatment of PrECs and a BPH cell line (BPH-1) that expresses the androgen receptor (AR) with the male sex hormone dihydrotestosterone (DHT) increased the basal levels of POP3 mRNA and protein, but not AIM2, and inhibited activation of the AIM2 inflammasome. Of interest, a stable knockdown of POP3 protein expression in the BPH-1 cell line increased cytosolic DNA-induced activation of AIM2 inflammasome. These observations suggest a potential role of POP3 protein in aging-related prostatic inflammation.

## 1. Introduction

Molecular mechanisms that contribute to the development of aging-related prostatic inflammatory diseases, including benign prostate hyperplasia (BPH), remain largely unknown [1,2,3,4]. Notably, studied using biopsies from patients with BPH and informative animal models have indicated a role for prostatic inflammation (PI) in the development of BPH [5,6,7,8,9].

Prostatic infections induce production of T1 interferons (IFN-α/β) through activation of the cGAS-STING pathway as a part of innate immune response [4,10,11]. The T1 IFNs, upon binding to a cell surface receptor, activate the JAK/STAT signaling in cells, resulting in stimulation of the expression of T1 IFN-inducible proteins [12]. The T1 IFN-inducible PYHIN protein family includes human IFI16 proteins, pyrin-only protein 3 (POP3), and AIM2 protein [13,14,15]. The proteins in the family share the *N*-terminal PYRIN domain (PYD) and the C-terminal HIN domain [13]. The PYD allows homotypic protein-protein interactions and the HIN domain allows sequence-independent binding to DNA [13,14]. The POP3 protein lacks the HIN domain [15].

We have reported earlier that treatment of human normal prostate epithelial cells (PrECs) and normal prostate stromal cells (PrSCs) with T1 IFN increased the levels of the androgen receptor (AR) and stimulated the transcription of AR-regulated gene [16]. Activation of AR in PrECs and PrSCs by the male sex hormone, dihydrotestosterone (DHT), regulates cell proliferation and survival [17,18]. Further, we noted earlier that activation of the AR in human prostate cancer cell line PC-3 stimulated the expression of IFI16 PYHIN proteins [19]. Increased expression of the IFI16 proteins in human normal PrECs, PC-3 prostate cancer cell line, and human normal diploid fibroblasts (HDFs) associated with the onset of cellular senescence [20,21,22,23]. Of interest, AR also drives human PrECs to cellular senescence [24]. Although the senescent cells exit the cell cycle permanently and do not divide, these cells secrete proteases and proinflammatory cytokines (e.g., IL-6, IL-1β, and IL-18) [25,26]. This phenotype of senescent cells has been termed senescence-associated secretory phenotype (SASP) and the phenotype is thought to contribute to aging-related chronic inflammation [25,26]. Notably, senescent PrECs accumulate in BPH [27] and their SASP promotes BPH [28]. However, the molecular mechanisms that contribute to the development of SASP in the senescent PrECs remain unclear.

The AIM2 protein senses cytoplasmic dsDNA in a variety of cell types and recruits an adaptor protein ASC through its PYD to form the AIM2 inflammasome [13,14]. The activated AIM2 inflammasome through activation of caspase-1 protease proteolytically cleaves the gasdermin D protein, pro-IL-1β, and pro-IL-18 [29]. Activated gasdermin D induces cell death by pyroptosis [29]. Proteolytic cleavage of pro-IL-1β, and pro-IL-18 promotes the secretion of the mature IL-1β and IL-18 proinflammatory cytokines [13,14,29]. Notably, increased levels of the POP3 protein in macrophages bound with PYD of AIM2 protein and the binding diminished the ability of the AIM2 protein to bind with ASC adaptor protein and to form AIM2 inflammasome [15].

Given that human prostatic infections are associated with chronic inflammation [4,5], and the development of BPH is associated with an accumulation of senescent PrECs with SASP [27,28], we investigated the role of POP3 and AIM2 proteins in senescent PrECs. Here we report that levels of the POP3 protein decreased in senescent PrECs as compared with pre-senescent proliferating or old cells. Further, DHT-mediated activation of the AR in human PrECs and in a benign prostate hyperplasia (BPH) cell line (BPH-1) up-regulated the expression of POP3 protein and inhibited cytosolic DNA-induced activation of the AIM2 inflammasome. Further, a knockdown of POP3 protein expression in BPH-1 cells activated the activity of the AIM2 inflammasome. Our observations have important implications for the development of aging-related prostatic inflammatory diseases.

## 2. Materials and Methods

### 2.1. Reagents

Synthetic double-stranded DNA (Poly (dA:dT)) in complex with transfection reagent (LyoVec) and LyoVec were from InvivoGen (San Diego, CA, USA) and EDTA-free protease inhibitor cocktail was from Roche Applied Science (Indianapolis, IN, USA). Dihydrotestosterone (DHT) was purchased from Sigma-Aldrich (St. Louis, MO, USA) and a stock (100 mM) was prepared in 100% ethanol and stored at −20 °C.

### 2.2. Primary PrECs, Prostate Cell Line, and Treatments

Human primary prostate epithelial cells (PrECs; at passage 2) from different donors (age 19 to 37) in culture (or frozen vials) were purchased from Lonza (Houston, TX, USA). Cells were maintained in culture as suggested by the supplier in the presence of medium supplements that were provided by the supplier as a part of the PrEGM™ Bulletkit™. Immortalized BPH-1 cell line was originally provided by Dr. Simon Hayward (Vanderbilt University Medical Center, Nashville, TN, USA) [30].

When indicated, cells in culture were treated with the indicated concentrations of dihydrotestosterone (DHT; stock in 100% ethanol) in phenol-free culture medium that was supplemented with charcoal/Dextran-treated fetal bovine serum (to decrease the endogenous levels of the steroid hormones) from the US source (from HyClone).

The asynchronous onset of cellular senescence in the primary cultures of human PrECs in late passages (passage 7 and higher) was assessed using well-described criteria for cellular senescence, including cell morphological changes and positivity to senescence-associated acidic β-galactosidase (SA-β-gal) as we have described [20]. In senescent cultures of PrECs, >90% cell population tested positive for the SA-β-gal, exhibited a large and flat cell morphology, and stopped cell proliferation [20].

### 2.3. Antibodies

Following antibodies were used to specifically detect proteins in immunoblotting: AR (sc-816), IFI16 (sc-8023), ASC (sc-22514), IL-1β (sc-7884), and IL-18 (sc-7954) from Santa Cruz Biotech (Santa Cruz, CA, USA); Caspase-1 (AHZ0082) from Invitrogen (Grand Island, NY, USA); Anti-STAT1 (cat # 9172), p-STAT1 (cat # 9171), and β-actin (cat # 4967) from Cell Signaling Technology (Danvers, MA, USA). Rabbit polyclonal antibodies that we raised against the C-terminal AIM2 peptide that specifically detected two human hAIM2 isoforms have been described [31]. Specific custom anti-peptide rabbit polyclonal antibodies were raised against a peptide (REEQETGICGSPSSARSV) in the POP3 protein, which detected an IFN-inducible POP3 protein of an expected size (~18 kDa) in total cell extracts from IFN-treated THP-1 cells as described [15]. Horseradish peroxidase (HRP) conjugated secondary anti-mouse (NXA-931) and anti-rabbit (NA-934) antibodies were from GE Healthcare Biosciences (Piscataway, NJ, USA).

### 2.4. Immunoblotting

Total cell lysates were prepared in radio-immunoprecipitation assay (RIPA) buffer (50 mM Tris-Cl (pH 7.4), 150 mM NaCl, 1% Nonidet P-40, 0.5% sodium deoxycholate, 0.1% sodium dodecyl sulfate) that was supplemented with complete mini EDTA-free protease inhibitor cocktail and phosphatase inhibitors (Cell Signaling, Danvers, MA, USA) as described [31]. The lysates containing approximately equal amounts of total proteins (~25−50 μg) were subjected to immunoblotting [31]. When indicated, actin protein was used as an internal control (because levels of actin did not change after DHT-treatment of cell types that we used). Enhanced chemiluminescence (ECL) signals of proteins were measured by the Molecular Imager Gel Doc XR^+^ System (Bio-Rad, Hercules, CA, USA). Immunoblots that were used for quantification of protein levels are shown in Supplementary Figures and the quantification values in an accompanied Table.

### 2.5. Inflammasome Assay

Activation of inflammasome activity in PrECs or BPH-1 cells was assessed using the criteria described earlier [32]. In brief, we subjected the total cell lysates or proteins from cell culture medium to immunoblotting and assessed (i) a decrease in the cellular levels of pro-caspase-1 (p45); (ii) an increase in the cellular levels of activated caspase 1 (p20) and/or (p10); (iii) a decrease in the cellular levels of pro-IL-1β (p31); and (iv) an increase in the cellular levels of the mature IL-1β (p17). Notably, in contrast to macrophages [32], in PrECs and BPH-1 cell line, activation of the inflammasome activity was accompanied by moderate to appreciable changes in the cellular levels of pro-caspase-1 (p45) and pro-IL-1β (p31) under our experimental conditions as described [31]. Further, when indicated, we detected the secreted levels of the mature IL-1β and IL-18 in the culture medium after precipitation of the proteins from the medium.

### 2.6. RNA Isolation and PCR

Cells were collected by centrifugation and the pellets were suspended into the Trizol reagent (Invitrogen) to isolate total RNA as described [31]. cDNA synthesis and semi-quantitative RT-PCR were performed as described [31]. The following primers were used for RT-PCR: the human AR (forward: 5′-CATCTGTGAAATAGAGCCTATCATATCCAC-3′; backward: 5′-TAACGCCTGCCTAGTGGCTTTGGAG-3′), IFI16 (forward: 5′-CCAAGACT GAAGAC TGAA-3′; backward: 5′-ATGGTCAATGACATCCAG-3′), POP3 (forward: 5′-ATGGAGA GTAAATATAAGGAG-3′; backward: 5′-TCAACATGCATTCCCA GAAAT-3′), AIM2 (forward: 5′-ATGTGAAGCCGTCCAGA-3′; backward: 5′-CATCATT TCTGATGG CTGCA-3′), and actin (forward: 5′-GCTCGTCGT CGACAACGGCTC-3′; backward: 5′-CATG ATCTG GGTCATCTTCTC-3′). Levels of actin mRNA were used as an internal control. To determine the fold change (FC) in the levels of an mRNA following a treatment, the intensity of the actin DNA band (an internal control) on the agarose gel and the DNA band of a gene of interest were measured by the Molecular Imager Gel Doc XR^+^ System (Bio-Rad, Hercules, CA, USA) with Image Lab Software. Next, the ratio was calculated using the DNA band intensity value for the gene of interest and actin DNA band. This ratio in control cells was indicated as 1 and the FC for DHT-treated samples was calculated by calculating the ratio between the value from treated samples (calculated as in the case of control sample) and the control value 1.

For quantitative real-time TaqMan PCR assays, Applied Biosystems’s (Foster City, CA, USA) technique was used [31]. The PCR cycling program consisted of denaturing at 95 °C for 10 min and 40 cycles at 95 °C for 15 s, and annealing and elongation at 60 °C for 1 min. The TaqMan assays for *IFI16* (assay Id #Hs00194216_mL), human interferon-β (*IFNB*; assay Id # Hs01077958 _s1), and for the endogenous control β-actin (assay Id# Hs99999903_mL) were purchased from Applied Biosystems (Foster City, CA, USA) and used as suggested by the supplier. The POP3 TaqMan assay was custom designed: (forward: 5′-AGCACGAGTAGCCAACTT GATT-3′; backward: 5′-GGTCTTCCTCACTGCAGACA-3′).

### 2.7. Transfection

Sub-confluent cultures of PrECs or BPH-1 cells were either treated with vehicle (ethanol) or with the indicated concentrations of DHT (in ethanol) as noted. Following the treatment, cells were “primed” with TNF-α for 3 has described [31]. Control or “primed” cells were either transfected with LyoVec (control) or poly (dA:dT)/LyoVec (5 μg/mL) for the indicated time. At the end of incubations, cells were harvested to prepare total cell lysates.

### 2.8. Stable Knockdown of POP3 Expression

To knockdown POP3 protein expression in BPH-1 cell line, cells were either transfected with an empty vector (pcDNA3.1) or pcDNA3.1-POP3(AS) plasmid (a PCR fragment was cloned in the multiple cloning site in the vector in the reverse orientation), thus, allowing the expression of an antisense mRNA. The transfected cells were selected using G418 (500 μg/mL) for two weeks and the G418-resistant colonies (>300 colonies) were pooled. To maintain cells in culture, a reduced concentration (250 μg/mL) of the G418 was used. The transfected cells were cultured without G418 in the medium for two days prior to the experiments.

### 2.9. Statistical Methods

Experiments involving immunoblotting and semi-quantitative RT-PCR techniques were repeated at least 3-times. A representative result is shown. For quantitative PCR, the assays were performed in triplicates. Fold-changes in the levels of certain proteins and mRNAs are indicated based on the quantitation of signal in independent experiments. The statistical measurement values, when indicated, were presented as means ± SEM. The statistical significance of differences in the measured mean frequencies between the two experimental groups was calculated using the Student two-tailed *t*-test.

## 3. Results

### 3.1. Activation of Type I Interferon Signaling in Senescent PrECs Differentially Regulated the Expression of POP3 and AIM2 Proteins

**Figure 1:**
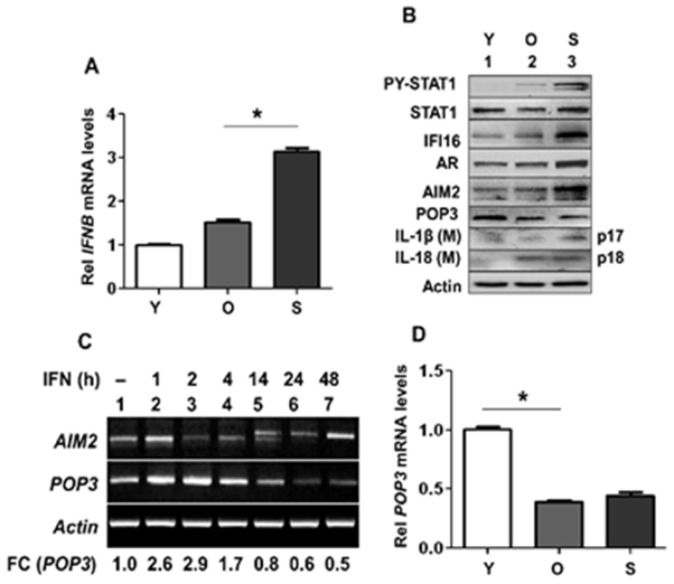
Activation of type I interferon signaling in human senescent PrECs differentially regulated the expression of POP3 and AIM2. (**A**) Total RNAs isolated from young proliferating (Y; passage-2), old (O; passage-5), or senescent (S; passage-8) human PrECs were subjected to quantitative real-time PCR using the TaqMan assay specific for the human *IFNB* mRNA. The RNA levels were normalized using *ACTIN* mRNA. The relative levels of the *IFNB* mRNA in young PrECs are indicated as 1. The values indicated as SEM (* *p* < 0.05). (**B**) Total cell extracts prepared from young (Y; passage-2), old (O; passage-5), or senescent (S; passage-8) human PrECs were analyzed by immunoblotting using the antibodies specific to the indicated proteins. The IL-1β(M) and IL-18(M) indicate the cleaved forms of the pro-IL-1β and pro-IL-18 that were detected in the culture medium. The experiments were repeated at least two times from cells derived from two different donors of different ages. Immunoblots that were used for quantification of protein levels are shown in Supplementary Figure S1 and the quantification of protein levels in an accompanied Table. (**C**) Young proliferating (Y; passage-2) PrECs were either left untreated or treated with 1000 u/mL of IFN-β for indicated times (h). Total RNA was isolated and subjected to RT-PCR for the indicated mRNAs as described in Material and Methods. Fold changes (FC) in the levels of POP3 mRNA were calculated as noted in Materials and Methods. (**D**) Total RNAs isolated from young, old, or senescent PrECs, as described in the panel (**A**), were subjected to the quantitative real-time PCR using the TaqMan assays (in triplicates) specific for the human *POP3* mRNA. The RNA levels were normalized using *ACTIN* mRNA. The relative levels of the *POP3* mRNA in young PrECs are indicated as 1. The values indicated as SEM (* *p* < 0.05).

Senescent human diploid fibroblasts (HDFs), as compared with young proliferating or old HDFs, expressed higher basal levels of the IFN-β and activated the type I IFN-signaling [22]. Further, activation of the type I IFN-signaling in senescent HDFs increased the levels of AIM2 protein but decreased IFI16 protein levels [22]. Therefore, we examined the expression of IFN-β and the IFN-β-inducible PYHIN-family proteins in proliferating (passage 2), old (passage 5), and senescent PrECs (passage 8). As shown in Figure 1A, the levels of IFN-β mRNA were significantly higher in senescent vs. proliferating or old PrECs. Consistent with our previous observations [22], senescent PrECs exhibited activation of T1 IFN response as compared with proliferating or old PrECs as determined by increases in the levels of type I IFN-inducible STAT1 protein and its activating phosphorylation on Tyr-701 residue (Figure 1B). Interestingly, in contrast to senescent HDFs, the levels of type I IFN-inducible IFI16 proteins were higher in senescent PrECs than the young or old PrECs. Expectedly [16], the levels of AR were also higher in senescent PrECs than the young or old PrECs. Further, the levels of AIM2 protein were higher in senescent vs. young or old PrECs. However, the levels of POP3 protein were lower in senescent vs. young or old PrECs. Because POP3 protein inhibited activation of the AIM2 inflammasome [15], we also examined the levels of the mature IL-1β (p17) and IL-18 (p18) in the culture medium. We found that the levels of IL-1β and IL-18 were higher in the culture media of the senescent PrECs than young proliferating cells. These observations thus suggested activation of an inflammasome in senescent PrECs.

IFN-β treatment of human macrophages for increasing length of time (0 to 48 h) differentially regulated the expression levels of POP3 and AIM2 mRNA [15]. Therefore, to investigate the potential role of AIM2 inflammasome activation in SASP of PrECs that activated the T1 IFN-signaling (Figure 1A), we treated proliferating PrECs with increasing length of time (0–48 h) and compared the levels of POP3 and AIM2 mRNA levels. As shown in Figure 1C, the treatment of PrECs with IFN-β increased the levels of AIM2 and POP3 mRNAs within an hour. However, the levels of AIM2 mRNA decreased after an hour of treatment but increased again after 48 h of treatment. In contrast, the levels of POP3 mRNA stayed higher after an hour of the treatment of PrECs but stayed lower after 14 h of treatment. Accordingly, a quantitative PCR revealed that old and senescent PrECs expressed lower basal levels of POP3 mRNA than proliferating cells (Figure 1D). These observations are consistent with a chronic activation of the T1 IFN-signaling in senescent PrECs, contributing to an increased AIM2/POP3 protein ratio through a transcriptional mechanism.

### 3.2. Androgen Receptor Activation in Proliferating PrECs Increased the Expression of POP3

**Figure 2:**
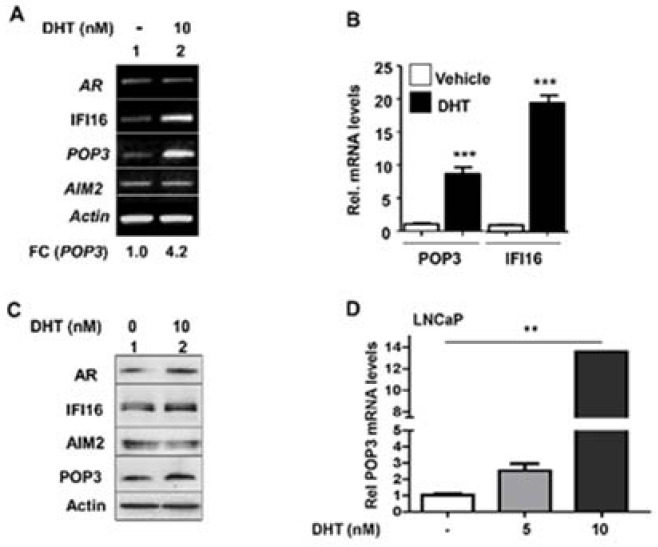
Androgen receptor activation in human proliferating PrECs increased the expression of POP3. (**A**) Sub-confluent cultures of proliferating young (passage 2) human PrECs were either treated with vehicle (ethanol) or 10 nM DHT for 18 h. After the treatment, total RNA was isolated and subjected to RT-PCR for the levels of mRNAs for the indicated genes. The fold change (FC) in the levels of POP3 mRNA in response to DHT-treatment of cells as compared with vehicle treated cells was estimated as described in methods. (**B**) Total mRNA isolated in panel (**A**) was subjected to quantitative real-time PCR (in triplicates) using the TaqMan assay specific for the indicated mRNA. The mRNA levels were normalized using *ACTIN* mRNA. The relative levels of the mRNA in vehicle treated PrECs are indicated as 1. The values indicated as SEM (* *p* < 0.05, ** *p* < 0.01, *** *p* < 0.001). (**C**) Cultures of young proliferating (passage 2) PrECs were either treated with vehicle (lane 1) or 10 nM DHT for 18 h as described in methods. After the treatment, total cell lysates containing equal amounts of proteins were subjected to immunoblotting using the antibodies specific to the indicated proteins. Immunoblots that were used for quantification of protein levels are shown in Supplementary Figure S2 and the quantification of protein levels in an accompanied Table. The experiment was repeated two times using cells derived from a single donor. (**D**) Cultures of the LNCaP cells were either treated with vehicle or the indicated concentration of DHT for 18 h. Total mRNA was isolated and subjected to quantitative real-time PCR using the TaqMan assay specific for the *POP3* mRNA. The *POP3* mRNA levels in all samples were normalized using *ACTIN* mRNA. The relative levels of the mRNA in vehicle treated LNCaP cells are indicated as 1. The values indicated as SEM (* *p* < 0.05, ** *p* < 0.01, *** *p* < 0.001).

Human primary PrECs express detectable levels of the androgen receptor (AR) [16]. Further, treatment of primary PrECs with type I IFN increased the levels of AR and stimulated the transcriptional activity of AR [16]. Because senescent PrECs exhibited activation of T1 IFN response and expressed higher basal levels of AR (Figure 1B), we tested whether activation of the AR in proliferating PrECs could regulate the expression of POP3 and AIM2. Consistent with our previous observations [19], treatment of proliferating PrECs with the male sex hormone DHT (10 nM) for 14 h increased the levels of *IFI16* mRNA (Figure 2A). Further, the treatment increased the levels of POP3 mRNA ~ 4-fold. However, the levels of AIM2 mRNA remain unchanged. Therefore, we performed quantitative PCR to assess the extent of increase in the levels of POP3 mRNA by DHT in PrECs. As shown in Figure 2B, treatment of cells with DHT significantly increased the levels of POP3 and IFI16 mRNAs. Accordingly, we also noted measurable increases in the levels of IFI16 and POP3 proteins in extracts from DHT-treated proliferating PrECs (Figure 2C). Consistent with these observations, treatment of LNCaP human prostate cancer cells, which express abundant levels of AR (as compared with normal proliferating PrECs) [33], with 10 nM concentration of DHT also increased the levels of POP3 mRNA (Figure 2D). Similarly, treatment of human benign prostate hyperplasia cell line BPH-1 with 10 nM DHT also increased the levels of IFI16 and POP3 proteins, but not AIM2 protein (data not shown). However, treatment of androgen independent human prostate cancer cell line PC-3 with 10 nM DHT did not increase the levels of POP3 protein (data not shown). Together, these observations are consistent with activation of T1 IFN-signaling in human senescent PrECs potentiating stimulation of the AR-mediated increases in the levels of IFI16 and POP3 proteins, but not the AIM2 protein.

### 3.3. Androgen Treatment of PrECs Inhibited Cytosolic DNA-Induced Activation of the AIM2 Inflammasome

**Figure 3:**
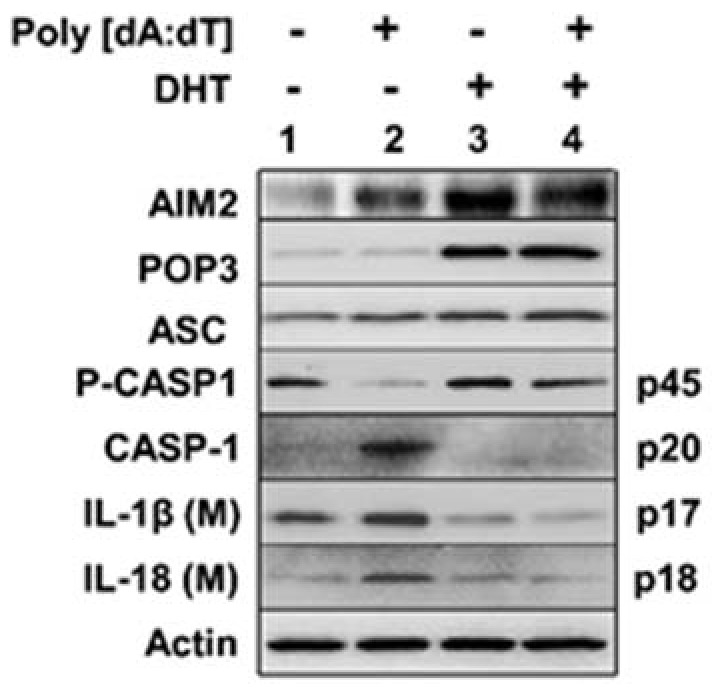
Androgen treatment of PrECs inhibited cytosolic DNA-induced activation of the AIM2 inflammasome. Cultures of human proliferating PrECs were either treated with vehicle (lanes 1 and 2) or with 10 nM DHT (lanes 3 and 4) for 18 h. Cells treated with either vehicle or DHT were further treated with 10 ng/mL TNF-α for 3 h to “prime” cells. The primed cells were either treated with LyoVec (lanes 1 and 3) or poly(dA:dT)/LyoVec (5 μg/mL; lanes 2 and 4) for 4 h. After the treatment, total cell lysates and culture medium (after precipitation of proteins) were subjected to immunoblotting using the antibodies specific to the indicated proteins. The IL-1β (M) and IL-18 (M) indicate the cleaved forms of the pro-IL-1β and pro-IL-18 that were detected in the culture medium. The experiment was repeated using proliferating PrECs from a single donor. Immunoblots that were used for quantification of protein levels are shown in Supplementary Figure S3 and the quantification in an accompanied Table.

To determine whether activation of androgen receptor in human proliferating PrECs, which increased the levels of POP3 protein (Figure 2), could inhibit AIM2 inflammasome activity, we compared the inflammasome activation in proliferating PrECs after vehicle (alcohol) or DHT treatment. As shown in Figure 3, DHT treatment of human primary PrECs (passage 2), as compared with control cells (vehicle treated), appreciably increased the levels of the POP3 protein (compare lane 3 with 1). Further, the basal levels of procaspase-1 (p45) were lower in control cells that were stimulated with the synthetic DNA poly [dA:dT] (compare lane 2 with 4). Accordingly, levels of the activated caspase-1 (p20) were higher in the control cells than DHT-treated cells that were stimulated with synthetic DNA. Similarly, the secreted IL-1β (p17) and IL-18 protein levels were higher in the culture medium of control cells than DHT-treated cells. Together, these observations indicated that activation of AR by DHT in proliferating normal PrECs up-regulated the levels of POP3 and the up-regulation associated with a decrease in cytosolic DNA-induced activation of the AIM2 inflammasome activity.

### 3.4. A Stable Knockdown of POP3 Protein Expression in BPH-1 Cell Line Increased Cytosolic DNA-Induced AIM2 Inflammasome Activation

**Figure 4:**
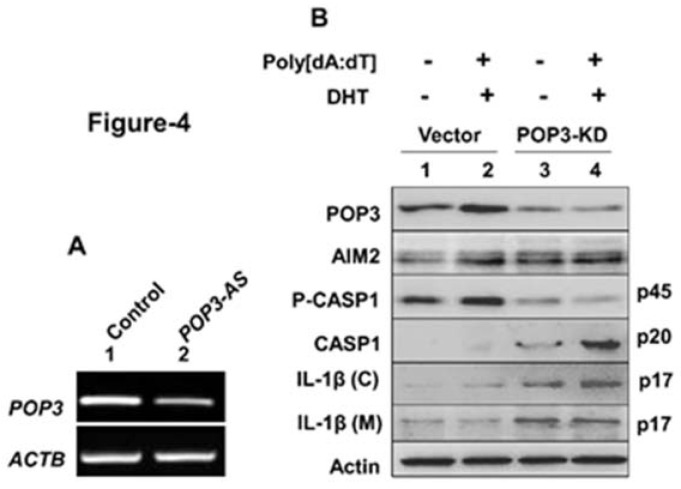
A stable knockdown of POP3 protein expression in BPH-1 cell line increased AIM2 inflammasome activation. (**A**) Total RNA isolated from vector transfected control BPH-1 cells (lane 1) or cells transfected with the pcDNA3.1-POP3(AS) vector allowing the expression of the antisense POP3 mRNA were analyzed by RT-PCR for the indicated genes. The experiment was repeated two times. (**B**) Control BPH-1 cells in panel (**A**) or cells transfected with pcDNA3.1-POP3(AS) vector were either treated with vehicle (lanes 1 and 3) or 10 nM DHT (lanes 2 and 4) for 18 h. Cells were further treated with 10 ng/mL TNF-α for 3 h to “prime” cells. The primed cells were incubated with either LyoVec (lanes 1 and 3) or poly (dA:dT)/LyoVec (5 μg/mL; lanes 2 and 4) for 4 h. After the treatment, total cell lysates and cell culture medium (after precipitation of proteins) were subjected to immunoblotting using the antibodies specific to the indicated proteins. The IL-1β (C), the cleaved IL-1β (p17) within the cell; IL-1β (M), cleaved IL-1β detected in the culture medium. The experiment was repeated two times. Immunoblots that were used for quantification of protein levels are shown in Supplementary Figure S4 and the quantification in an accompanied Table.

We also investigated whether a knockdown of POP3 protein expression in BPH-1 cells could increase activation of the AIM2 inflammasome without or after DHT treatment. As shown in Figure 4A, stable transfection of BPH-1 cells with an expression vector that allowed the expression of the antisense POP3 mRNA appreciably reduced the basal levels of POP3 mRNA in cells as compared with cells that were transfected with an empty vector. Further, androgen-treatment of vector transfected control cells and their stimulation with cytosolic synthetic DNA did not result in appreciable activation of the inflammasome activity as determined by the lack of detection of proteolytically cleaved caspase-1 (p20) in cell lysates and secreted mature IL-1β (p17) and IL-18 (p18) in the culture medium (Figure 4B). However, a knockdown of the POP3 protein expression in cells and their treatment with DHT did not result in a measurable increase in POP3 protein levels. Importantly, treatment of cells with the synthetic DNA robustly activated the inflammasome activity in cells, as measured by increases in the proteolytically cleaved and activated caspase-1 (p20) levels in cell lysates and the secreted levels of the mature IL-1β (p17) and IL-18 (p18) in the culture medium. Together, these observations indicated that a stable knockdown of POP3 protein expression in BPH-1 cell line activated cytosolic DNA-induced activity of the AIM2 inflammasome.

## 4. Discussion

Senescent PrECs accumulate in BPH [27]. Further, SASP is associated with activation of an inflammasome activity and an increase in the production of proinflammatory cytokines [34,35]. Therefore, our observations that (i) human senescent PrECs expressed higher basal levels of the AIM2 protein and lower basal levels of POP3 protein (Figure 1); and (ii) reduced basal levels of the POP3 protein in senescent PrECs (Figure 1) associated with activation of cytosolic DNA-responsive AIM2 inflammasome are consistent with a role of POP3 protein in the suppression of SASP in senescent PrECs.

Serum androgen levels decrease in men with aging [36,37]. Further, androgen receptor levels increase in PrECs in certain parts of the prostate [37]. Therefore, our observations that senescent PrECs that activated type I IFN signaling expressed higher basal levels of the AR (Figure 1B) and activation of androgen receptor in human PrECs increased the levels of POP3 protein (Figure 2) and increased levels of POP3 in PrECs inhibited cytosolic DNA-induced activation of the AIM2 inflammasome activity (Figure 3) support the idea that aging-related reduced serum levels of androgens in men contribute to a decrease in the levels of POP3 protein in PrECs, thus leading to an increase in activation of the AIM2 inflammasome. Because our observations implicate a role for the POP3 protein in aging-related prostatic inflammation, further work will be needed to examine the role of androgen-AR/POP3/AIM2 axis in the development of aging-related prostatic diseases.

POP3 protein also bound with the IFI16 proteins and inhibited activation of the IFI16 inflammasome [15]. Notably, androgens mediated activation of AR in human normal proliferating PrECs also increased the expression of *IFI16* gene [19]. As increased levels of IFI16 proteins in human proliferating PrECs potentiated the p53-mediated cell cycle arrest that is associated with cellular senescence [23], it is conceivable that androgens-mediated up-regulation of the POP3 protein in human PrECs also affects the cell cycle inhibitory functions of the IFI16 proteins. Because AR also drives human PrECs to cellular senescence [24], further work is needed to determine whether androgens-mediated increased levels of POP3 protein in human PrECs modulate the p53-mediated functions.

Activation of certain inflammasomes contributes to the development of prostatic diseases in animal models and humans [38,39,40,41]. These diseases include chronic prostatitis and chronic pelvic pain syndrome [38], BPH associated prostatic inflammation [39], and prostate cancer [40,41]. However, it remains unclear whether androgens-mediated activation of the AR in PrECs regulates the activity of the inflammasomes. Therefore, our observations that activation of AR in human PrECs suppressed activation of the AIM2 inflammasome are likely to serve the basis for further studies.

Androgen deprivation therapy (ADT) in prostate cancer patients is often associated with increased production of pro-inflammatory cytokines (e.g., IL1β) [28]. However, it remains unknown whether ADT in prostate cancer patients promote prostatic inflammation through activation of inflammasomes. Therefore, our observations that activation of AR in human PrECs up-regulated the expression of POP3 protein, an inhibitor of the production of inflammatory cytokines (IL-1β and IL-18) through activation of inflammasomes, are of significance.

The 5′-regulatory region of the *POP3* gene remains uncharacterized. Although the expression of *POP3* gene is induced by type I IFN [15], the IFN-responsive *cis*-element(s) remain unknown. Therefore, our observations that treatment of human PrECs (and BPH-1) cell line with androgen DHT increased levels of the POP3 mRNA and protein will require further work to identify the molecular mechanisms through which AR activation in PrECs increases the levels of POP3 mRNA and protein.

In summary, our observations identify the IFN-inducible POP3 PYHIN protein as a potential negative regulator of the AIM2 inflammasome and SASP in human senescent PrECs. These observations also suggest that aging-related reduced levels of androgens in men through reduced basal activation of the AR in PrECs increase the activation of the AIM2 inflammasome. Thus, our observations have important implications for aging-related development of prostatic inflammatory diseases.

## Data Availability

Not applicable.

## Supplementary Materials

The following are available online at https://www.mdpi.com/article/10.3390/life11040366/s1, Figure S1: Protein levels in immunoblots in Figure 1B were quantified and fold changes in protein levels were calculated as noted in the Materials and Methods. The table shows the values for the protein band intensity fractions using the GelQuant.NET software. The values in the parenthesis in the table indicate fold changes in protein levels, Figure S2: Protein levels in immunoblots in Figure 1C were quantified and fold changes in protein levels were calculated as noted above. The values in the parenthesis in the table indicate fold changes in protein levels, Figure S3: Protein levels in immunoblots in Figure 3 were quantified and fold changes in protein levels were calculated as noted above. The values in the parenthesis in the table indicate fold changes in protein levels, Figure S4: Protein levels in immunoblots in Figure 4B were quantified and fold changes in protein levels were calculated as noted above. The values in the parenthesis in the table indicate fold changes in protein levels.
