# Supplementary material for: Human Prostate Epithelial Cells Activate the AIM2 Inflammasome upon Cellular Senescence: Role of POP3 Protein in Aging-Related Prostatic Inflammation
Source: Life (Basel). 2021 Apr 20;11(4):366. doi: 10.3390/life11040366 (PMC8073538; doi:10.3390/life11040366)
Supplement: Supplementary file 1 [file life-11-00366-s001.zip › life-1106460-supplementary.pdf]

life

MDPI

Supplemental material

Human Prostate Epithelial Cells Activate the AIM2 Inflammasome upon Cellular Senescence: Role of POP3 Protein in Aging-Related Prostatic Inflammation

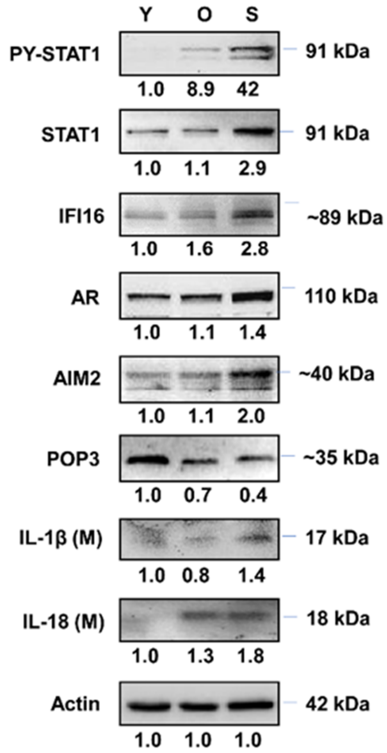

Supplementary Table

Protein	Protein band intensity (Fold change)		
	Young	Old	Senescent
PY-STAT1	0.00831(1)	0.07421(8.9)	0.35362(42)
STAT1	0.10386(1)	0.11437(1.1)	0.30267(2.9)
IFI16	0.18471(1)	0.29423(1.6)	0.52107(2.8)
AR	0.28565(1)	0.30723(1.1)	0.40712(1.4)
AIM2	0.24412(1)	0.26629(1.1)	0.48959(2.0)
POP3	0.45677(1)	0.32252(0.7)	0.22071(0.4)
IL-1β (M)	0.34431(1)	0.28647(0.8)	0.36922(1.4)
IL-18	0.17353(1)	0.2362(1.3)	0.59027(3.4)
Actin	0.15372(1)	0.14599(0.94)	0.15934(1.0)

Figure S1. Protein levels in immunoblots in Fig. 1B were quantified and fold changes in protein levels were calculated as noted in the Materials and Methods. The table shows the values for the protein band intensity fractions using the GelQuant.NET software. The values in the parenthesis in the table indicate fold changes in protein levels.

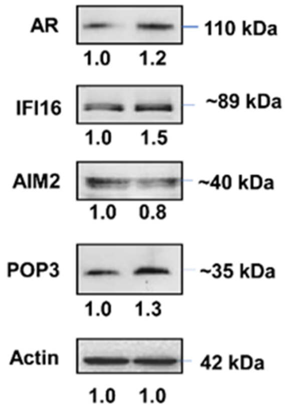

Supplementary Table

Protein	Protein band intensity (Fold change)	
	Control	DHT
AR	0.47612(1)	0.52388(1.2)
IFI16	0.4695(1)	0.6573(1.5)
AIM2	0.56563(1)	0.43437(0.8)
POP3	0.47147(1)	0.61291(1.3)
Actin	0.32491(1)	0.33387(1.0)

Figure S2. Protein levels in immunoblots in Fig. 1C were quantified and fold changes in protein levels were calculated as noted above. The values in the parenthesis in the table indicate fold changes in protein levels. .

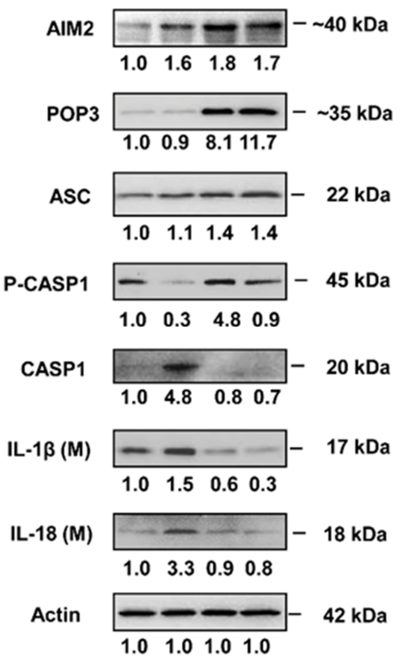

Supplementary Table

Protein	Protein band intensity (Fold change)			
Lane	1	2	3	4
AIM2	0.11851(1)	0.18864(1.6)	0.23186(1.8)	0.21025(1.7)
POP3	0.02475(1)	0.02407(1.0)	0.20184(8.1)	0.29046(11.7)
ASC	0.06697(1)	0.07429(1.1)	0.09662(1.4)	0.09795(1.4)
P-CASP1	0.18328(1)	0.04863(0.3)	0.29688(4.8)	0.17608(0.9)
CASP1	0.1351(1)	0.6572(4.8)	0.1087(0.8)	0.099(0.7)
IL-1β (M)	0.24853(1)	0.36813(1.5)	0.14932(0.6)	0.9432(0.3)
IL-18 (M)	0.07776(1)	0.25978(3.3)	0.07406(0.9)	0.06256(0.8)
Actin	0.26256(1)	0.27154(1)	0.25988(1)	0.25738(1)

Figure S3. Protein levels in immunoblots in Fig. 3 were quantified and fold changes in protein levels were calculated as noted above. The values in the parenthesis in the table indicate fold changes in protein levels.

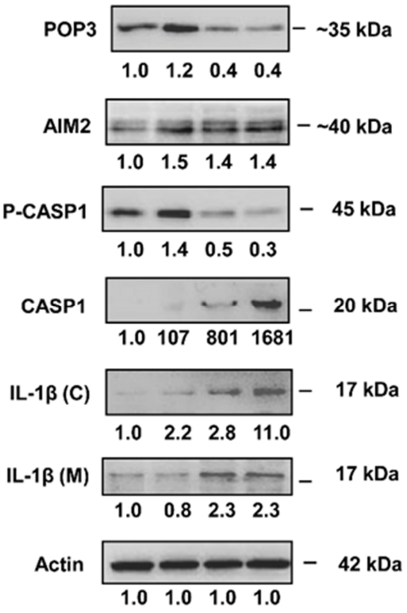

Supplementary Table

Protein	Protein band intensity (Fold change)			
Lane	1	2	3	4
POP3	0.2329(1)	0.2852(1.2)	0.1102(0.4)	0.0923(0.4)
AIM2	0.019(1)	0.2924(1.5)	0.2592(1.4)	0.2585(1.4)
P-CASP1	0.3106(1)	0.4274(1.4)	0.161(0.5)	0.101(0.3)
CASP1	0.0003(1)	0.0322(107)	0.2404(801)	0.5044(1681)
IL-1β (C)	0.0409(1)	0.0927(2.2)	0.1182(2.8)	0.4532(11)
IL-1β (M)	0.0843(1)	0.0752(0.8)	0.201(2.3)	0.1946(2.3)
Actin	0.1081(1)	0.1064(1)	0.108(1)	0.1098(1)

Figure S4. Protein levels in immunoblots in Fig. 4B were quantified and fold changes in protein levels were calculated as noted above. The values in the parenthesis in the table indicate fold changes in protein levels.
